# Automatic speech-based assessment to discriminate Parkinson’s disease from essential tremor with a cross-language approach

**DOI:** 10.1038/s41746-024-01027-6

**Published:** 2024-02-17

**Authors:** Cristian David Rios-Urrego, Jan Rusz, Juan Rafael Orozco-Arroyave

**Affiliations:** 1 GITA Lab, Faculty of Engineering, University of Antioquia, Medellín, Colombia; 2 Department of Circuit Theory, Czech Technical University in Prague, Prague, Czech Republic; 3 Pattern Recognition Lab, Friedrich-Alexander-Universität Erlangen-Nürnberg, Erlangen, Germany

**Keywords:** Diseases, Diagnostic markers, Neurological disorders

## Abstract

Parkinson’s disease (PD) and essential tremor (ET) are prevalent movement disorders that mainly affect elderly people, presenting diagnostic challenges due to shared clinical features. While both disorders exhibit distinct speech patterns—hypokinetic dysarthria in PD and hyperkinetic dysarthria in ET—the efficacy of speech assessment for differentiation remains unexplored. Developing technology for automatic discrimination could enable early diagnosis and continuous monitoring. However, the lack of data for investigating speech behavior in these patients has inhibited the development of a framework for diagnostic support. In addition, phonetic variability across languages poses practical challenges in establishing a universal speech assessment system. Therefore, it is necessary to develop models robust to the phonetic variability present in different languages worldwide. We propose a method based on Gaussian mixture models to assess domain adaptation from models trained in German and Spanish to classify PD and ET patients in Czech. We modeled three different speech dimensions: articulation, phonation, and prosody and evaluated the models’ performance in both bi-class and tri-class classification scenarios (with the addition of healthy controls). Our results show that a fusion of the three speech dimensions achieved optimal results in binary classification, with accuracies up to 81.4 and 86.2% for monologue and /pa-ta-ka/ tasks, respectively. In tri-class scenarios, incorporating healthy speech signals resulted in accuracies of 63.3 and 71.6% for monologue and /pa-ta-ka/ tasks, respectively. Our findings suggest that automated speech analysis, combined with machine learning is robust, accurate, and can be adapted to different languages to distinguish between PD and ET patients.

## Introduction

Essential tremor (ET) is a syndrome characterized by an isolated bilateral upper-limb action tremor with a duration of at least 3 years, with or without signs in other body parts, such as head, larynx (voice tremor), or lower limbs^1^. In contrast, Parkinson’s disease (PD) is caused by the progressive loss of dopaminergic neurons in the substantia nigra of the midbrain and is characterized by rigidity, bradykinesia, and postural instability, among other symptoms^2^. Both PD and ET share tremor as a common clinical feature. However, PD is characterized by resting tremor, which occurs when muscles are relaxed, while action tremor during voluntary muscle contraction is an early sign of ET. The onset of ET may also be accompanied by the presence of resting tremor. Both disorders can also exhibit non-motor symptoms such as cognitive impairments, sleep disturbances, depression, and anxiety. Therefore, the differential diagnosis of these disorders is challenging for clinicians due to their overlapping symptoms^3,4^. Misdiagnoses occur mainly in the early stages of the disease when clinical signs are subtle. Previous studies have noted that one-third of patients diagnosed with ET had been previously misdiagnosed with PD^5,6^.

Movement disorders such as PD and ET typically co-occur with two types of distinct dysarthria that reflect the underlying pathophysiology: hypokinetic and hyperkinetic. It should be noted, however, that these disorders can sometimes exhibit mixed features^7^. Hypokinetic dysarthria is typically present in PD, and its characteristics include monoloudness, monotonicity, imprecise pronunciation of consonants and vowels, and lack of fluency, among other symptoms^8^. In contrast, ET is typically associated with hyperkinetic dysarthria, which generally arises from involuntary movements associated with tremor. In such cases, the most relevant speech deficits include phonatory and prosodic disturbances that are primarily caused by tremor^9^. However, evidence of speech dysfunction in both disorders is mainly based on single language assessment. Scenarios where different languages are adapted to perform clinical assessments are underexplored.

Previous studies have focused on differentiating between PD and ET in patients through the use of different sources of information, including video-taped neurological examinations^10^, hand tremor signals^11^, gait signals^12^, electromyogram signals^13^, handwriting signals^14^, and medical images^15^. Although previous studies have shown that it is possible to differentiate the hypokinetic dysarthria in PD from the hyperkinetic dysarthria of disorders such as Huntington’s disease^4,7,16,17^, the speech-based differentiation between PD and ET has never been investigated. The potential of speech-based differentiation between PD and ET should first be determined in patients with a definitive clinical diagnosis, with the future goal of evaluating speech analysis as a diagnostic instrument in early-stage differentiation.

However, there is a distinct lack of databases that can be used to develop frameworks for the differentiation of neurodegenerative diseases based on speech assessment and machine learning. These limitations are even more pronounced due to the different standardizations used for voice recordings, which include the type of microphone used as well as acoustic conditions^7^. In particular, phonetic variability across different languages imposes considerable practical challenges for developing a unified speech assessment framework^18^. This has motivated the scientific community to explore the possibility of adapting information from different languages to assess certain pathologies^19^, which has raised questions about informational deficits in language-dependent speech dimensions and features^20^. Indeed, some studies have shown that differences in language did not impact the clinical assessment of disease phenotypes^18,21^. Therefore, the development of cross-languages and/or cross-pathology models could be the way to find robust models, with high performance and sufficient generalization for voice-based pathology classification and monitoring. In this context, the differentiation between PD and ET can provide a unique theoretical model for the testing of such a framework, which could potentially be clinically applied to the early diagnosis of diseases with similar clinical manifestations.

This paper introduces a model where information from different languages is adapted for the automatic classification of PD and ET using speech signals. Here, we propose a classical approach using a Gaussian mixture model-universal background model (GMM-UBM) and support vector machines (SVM) for the domain adaptation of both language and pathology, considering different recording parameters such as microphones, acoustic conditions, and protocols between the databases. On the one hand, GMM-UBM allows performing knowledge transfer from GMMs per speech dimension (articulation, phonation, and prosody) with the main advantage of being interpretable and enabling the relation of different symptoms associated with the disease; furthermore, considering the data scarcity scenario, this method is the best choice. The primary hypothesis is that a UBM trained on data from utterances produced by speakers who speak specific languages can be used to model speech impairments in PD and ET patients who speak a different language; in other words, data trained to differentiate between PD and healthy speech in German or Spanish can be used to evaluate speech impairments in Czech. SVM allows the training and evaluation of models with small amounts of data using linear functions in high-dimensional feature space, resulting in the generation of robust and generalized models that can be used to discriminate between patients and controls. Specifically, we aimed to assess: (1) how effectively can models be used to distinguish between PD and ET patients using speech signals, (2) which dimensions of speech are most greatly affected between pathologies, and (3) which language shows greater compatibility in the proposed methodology.

## Results

Two different experiments were performed in this study, with both experiments using data from the Czech corpus: (A) PD patients vs. ET patients; and (B) healthy control (HC) subjects vs. PD patients vs. ET patients. The Czech speaker adaptation was based on the UBM models created with recordings from Spanish and German datasets, and a combination of both. We then obtained GMM supervectors for each speech dimension (i.e., articulation, phonation, and prosody) for each UBM model. Additionally, the fusion of the three speech dimensions and dimensionality reduction of the fusion by using principal component analysis (PCA) were considered. This last scheme was only performed on the fusion of the three speech dimensions, and the number of components was determined by 90% of the cumulative variance.

We initially considered creating different UBM models for control subjects, patients, and a combination of the two groups. However, all classification models that used patients during UBM training yielded lower performances. Consequently, we only reported UBM models generated from samples of HC subjects. We hypothesize that the inclusion of patients in the UBMs resulted in highly variable models; these models tended to be unstable and were thus unsuitable for further analyses. Another possible reason is that the number of subjects was not large enough to “cover” the high variability observed when the patient data were aggregated due to the wide variety of abnormal patterns that arose from dysarthric symptoms.

Due to the aforementioned reasons, we considered creating an additional three UBM models with larger numbers of Spanish and German recordings using the CIEMPIESS (Spanish) and Verbmobil (German) datasets; a UBM was also trained from a combination of these two datasets. These corpora were added to determine if the amount of data used to train the UBMs affected the adaptation process of the target samples.

### Bi-class classification: PD patients vs. ET patients

Table 1 shows the accuracy obtained from the classification of PD patients vs. ET patients using each speech dimension, their fusion, and their dimension reduction. The accuracy of the models ranged between 60–80%. The best result was obtained from the /pa-ta-ka/ task using a supervector built with a fusion of the three speech dimensions adapted from the UBM trained using controls from the German databases. This approach yielded an accuracy of 86.2% and a good balance between sensitivity (87.6%) and specificity (84.8%). For the case of the monologue task, we obtained an accuracy of 81.4%, with a sensitivity of 83.2% and a specificity of 79.6%. When each speech dimension was analyzed separately, it was found that the articulation dimension performed the best (accuracy of 72.3 ± 1.7%) when using the monologue task, while prosody outperformed the other dimensions when the /pa-ta-ka/ repetitions were assessed (accuracy of 78.3 ± 0.4%).

**Table 1:** Bi-class classification: PD patients vs. ET patients with each speech dimension and their fusion

UBM	Monologue								Pataka							
	Articulation		Phonation		Prosody		Fusion	PCA	Articulation		Phonation		Prosody		Fusion	PCA
	M	Acc. (%)	M	Acc. (%)	M	Acc. (%)	Acc. (%)	Acc. (%)	M	Acc. (%)	M	Acc. (%)	M	Acc. (%)	Acc. (%)	Acc. (%)
German	4	70.8 ± 1.6	32	68.2 ± 2.2	8	58.0 ± 2.5	77.2 ± 3.2	47.6 ± 4.0	2	69.0 ± 2.3	64	70.4 ± 2.0	2	79.0 ± 0.3	**86.2** **±** **1.2**	80.6 ± 1.0
Spanish	4	73.8 ± 1.3	16	69.8 ± 1.9	2	60.2 ± 3.8	75.4 ± 1.9	53.6 ± 1.5	2	71.4 ± 2.7	8	65.6 ± 2.4	2	78.2 ± 0.4	84.6 ± 0.5	75.4 ± 1.7
German-Spanish	4	71.6 ± 2.4	32	67.4 ± 3.4	4	54.0 ± 2.2	74.8 ± 1.2	46.2 ± 1.9	2	70.8 ± 2.5	16	65.0 ± 3.2	2	77.8 ± 0.4	82.2 ± 1.2	79.4 ± 2.0
CIEMPIESS (Spanish)	2	69.4 ± 1.4	64	66.6 ± 1.4	2	60.6 ± 2.7	77.4 ± 1.4	55.6 ± 1.6	–	–	–	–	–	–	–	–
Verbmobil (German)	4	73.6 ± 2.7	64	71.0 ± 1.6	8	62.2 ± 4.0	**81.4** **±** **1.7**	73.8 ± 5.7	–	–	–	–	–	–	–	–
CIEMPIESS-Verbmobil	4	74.4 ± 1.0	32	75.8 ± 1.9	8	59.6 ± 2.1	81.2 ± 1.2	41.2 ± 3.2	–	–	–	–	–	–	–	–
Average	–	72.3 ± 1.7	–	69.8 ± 2.1	–	59.2 ± 2.9	77.9 ± 1.8	48.0 ± 3.0	–	70.4 ± 2.5	–	67.0 ± 2.5	–	78.3 ± 0.4	84.3 ± 0.9	78.5 ± 1.6

*Acc* accuracy, *M* number of Gaussian components. mean ± standard deviation.

**Fig. 1:**
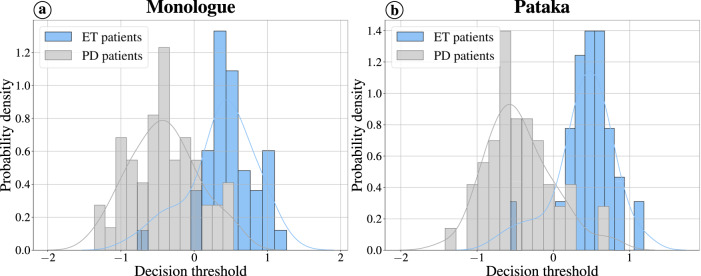
Histograms and the corresponding probability density distributions of the scores obtained in the best classification scenarios between PD and ET patients in Czech. **a** For the monologue task, the adaptation was performed from the UBM trained with Verbmobil (German). **b** For the /pa-ta-ka/ task from the UBM trained with the German controls, both scenarios were obtained with the fusion of the three speech dimensions.

Figure 1 shows the histograms and the probability density distributions of the scores obtained when classifying the samples i.e., the distance to the SVM hyperplane. The left side shows the result obtained from the monologue task using a fusion of the dimensions (accuracy: 81.4%), while the right side shows the best result obtained from a model trained on the /pa-ta-ka/ task (accuracy: 86.2%). Note that the errors are evenly distributed between the two classes.

### Tri-class classification: PD patients vs. ET patients vs. HC subjects

This approach used the same experiments as in the previous classification except that the models also attempted to classify speech from HC subjects, i.e., this is a tri-class classification problem. A one-vs-rest SVM was used to perform the classification. As in previous experiments, all results were assessed using the accuracy metric. Table 2 shows the results of these experiments. It is possible to observe that, as in the previous experiments, the best result for the monologue task was obtained from a fusion of the three speech dimensions using the supervector adapted from the Verbmobil (German) UBM (accuracy: 63.3%). For the /pa-ta-ka/ task, the best result was obtained using a supervector of the prosody dimension (accuracy: 71.6%). Once again, the controlled /pa-ta-ka/ task exhibited better performances than spontaneous speech. Furthermore, it was found that the fusion of speech dimensions allowed for the differentiation between PD patients, ET patients, and healthy speech with an average accuracy of 57.6 ± 1.7% regardless of the UBM from which the adaptation was performed. While for a diadochokinetic task, prosody is sufficient to discriminate between both disorders as well as healthy speech with a superior performance of 70.7 ± 1.8%.

**Table 2:** Tri-class classification: PD patients vs. ET patients vs. healthy speech with each speech dimension and their fusion

UBM	Monologue								Pataka							
	Articulation		Phonation		Prosody		Fusion	PCA	Articulation		Phonation		Prosody		Fusion	PCA
	M	Acc. (%)	M	Acc. (%)	M	Acc. (%)	Acc. (%)	Acc. (%)	M	Acc. (%)	M	Acc. (%)	M	Acc. (%)	Acc. (%)	Acc. (%)
German	4	57.5 ± 3.0	2	56.7 ± 1.9	4	50.4 ± 2.2	58.4 ± 1.8	29.5 ± 2.3	2	49.7 ± 1.1	32	55.9 ± 1.5	2	69.7 ± 2.0	68.0 ± 1.5	64.1 ± 2.2
Spanish	2	52.4 ± 2.6	8	56.9 ± 2.3	2	45.7 ± 1.1	57.2 ± 2.0	32.1 ± 1.4	4	50.5 ± 1.7	8	53.7 ± 1.6	2	70.8 ± 0.8	67.9 ± 2.6	68.1 ± 2.4
German-Spanish	4	54.3 ± 3.3	2	52.5 ± 1.5	4	50.5 ± 1.1	57.6 ± 0.7	30.1 ± 2.5	4	50.3 ± 2.8	2	56.8 ± 1.3	2	**71.6** **±** **2.5**	68.3 ± 1.9	67.2 ± 2.0
CIEMPIESS (Spanish)	2	58.8 ± 1.9	32	53.9 ± 2.0	4	50.2 ± 1.3	54.0 ± 2.0	27.7 ± 3.7	–	–	–	–	–	–	–	–
Verbmobil (German)	4	59.1 ± 1.5	64	55.5 ± 2.2	8	49.1 ± 1.7	**63.3** **±** **2.1**	27.9 ± 2.9	–	–	–	–	–	–	–	–
CIEMPIESS-Verbmobil	2	54.1 ± 2.0	16	56.0 ± 2.7	4	50.4 ± 1.7	55.2 ± 1.3	30.3 ± 2.4	–	–	–	–	–	–	–	–
Average	–	53.0 ± 2.4	–	55.3 ± 2.1	–	49.4 ± 1.5	57.6 ± 1.7	29.6 ± 2.5	–	50.2 ± 1.9	–	55.5 ± 1.5	–	70.7 ± 1.8	68.1 ± 2.0	66.5 ± 2.2

*Acc* accuracy, M number of Gaussian components. mean ± standard deviation.

**Fig. 2:**
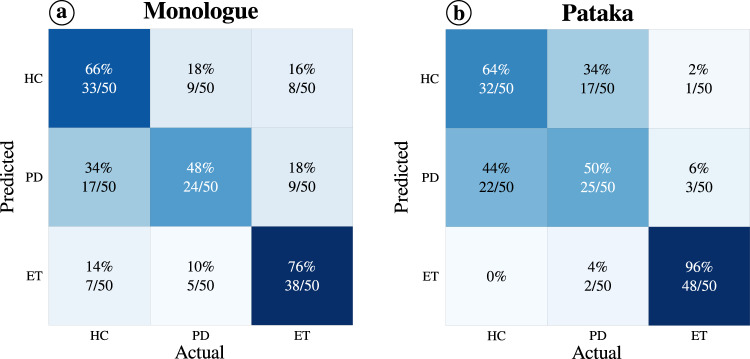
Confusion matrices of the best results obtained in the classification of ET patients (ET) vs. PD patients (PD) vs. healthy speech (HC). **a** For the monologue task, the adaptation was performed from the UBM trained with Verbmobil (German) and using the fusion of the three speech dimensions. **b** For the /pa-ta-ka/ task, the adaptation was performed from the UBM trained with the German and Spanish controls and using the prosody dimension.

Figure 2 shows the confusion matrices of the best results obtained for the two speech tasks. The monologue task yields an accuracy of 76% with ET patients, while only 48% of the PD subjects were correctly classified. Regarding the healthy controls, 66% of them were correctly classified. It should be noted that 34% of the PD subjects were misclassified as healthy controls. In the confusion matrix corresponding to the /pa-ta-ka/ task, a total of 48 of the 50 ET patients were correctly classified (accuracy: 96%), while 64% of healthy subjects were correctly classified (most of the incorrectly classified healthy subjects were classified as PD patients), and finally, 50% of the patients with PD were correctly classified. This result shows that prosody (which was the most discriminative speech dimension for this experiment) models the characteristic tremor of ET patients and allows for their discrimination against PD patients and healthy controls. In addition, to determine if there was any correlation between PD patients classified as HC subjects and their severity level (e.g., were patients at an early stage of the disease more commonly misclassified as HC subjects?), we performed a Mann–Whitney *U*-test on the UPDRS-III scores of PD patients classified as HC subjects and correctly classified patients. We obtained a *p* value of 0.374 for PD patients who were correctly predicted by the monologue task (*n* = 24; UPDRS-III mean = 19.8, SD = 10.1) compared to those were had been misclassified (*n* = 17; UPDRS-III mean = 16.7, SD = 7.3). For the /pa-ta-ka/ task, we report a *p* value of 0.203 between the correctly classified PD patients (*n* = 25; UPDRS-III mean = 21.7, SD = 9.6) compared to the misclassified PD patients (*n* = 22; UPDRS mean = 17.7, SD = 9.4). Therefore, we can conclude that there is no significant difference in the severity level of PD patients correctly classified and misclassified in both tasks.

**Fig. 3:**
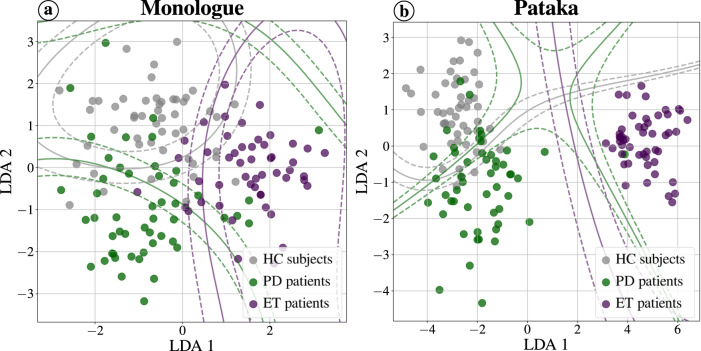
Visualization of the groups distribution after applying LDA using two components. **a** Results based on monologue task. **b** Results based on /pa-ta-ka/ task.

Figure 3 shows the distribution of speakers in each group based on the best results from Table 2. This representation was created by concatenating the three supervectors of articulation, phonation, and prosody for the monologue task as well as the prosody supervector for the /pa-ta-ka/ task. The original space was reduced to two dimensions by applying linear discriminant analysis (LDA). Three clusters can be observed in the figure corresponding to the monologue task (Fig. 3a) (one for each class); however, it is clear that some samples overlap with each other, consistent with our results and the presented confusion matrix. In contrast, the accurate discrimination of ET patients is clearly observed for the /pa-ta-ka/ task (Fig. 3b), although there is a distinct overlap between healthy subjects and PD patients. These results are consistent with the trends shown in Fig. 2.

Finally, to evaluate whether each representation contained information on the age of participants and/or the severity level of the patients, we computed the Spearman correlation between each component, the age of the patients, and their severity level (UPDRS-III and TETRAS scores for PD and ET, respectively). The results showed that, in most scenarios, there was no correlation (*ρ* = 0), while some scenarios exhibited very weak correlations ($0 < \left\vert \rho \right\vert \le 0.2$) between the LDA components and the age or motor severity of the patients. No significant differences were observed in any of the methods (*p* value >0.05). It is important to note that the PD/ET/HC groups were well-balanced in terms of the age of the subjects; therefore, age should not be a confounding factor in the analysis.

## Discussion

We found that the models achieved PD and ET classification accuracies of up to 86% in a controlled task (i.e., /pa-ta-ka/) and 81% in a spontaneous speech task (i.e., monologue). Both tasks were modeled using the three speech dimensions: articulation, phonation, and prosody. The results suggest that the three dimensions are complementary, with each contributing to the highly accurate classification results. We believe that this finding is promising and could potentially be used to support the differential diagnosis between PD and ET. When each dimension was analyzed separately using the monologue task, it was found that articulation had the strongest contribution to discrimination accuracy. This finding is most likely explained by the fact that PD patients with hypokinetic dysarthria are characterized by rigidity of the muscles involved in the speech production process and that this phenomenon is most accurately modeled by the transition between voiced and unvoiced segments (articulation)^22^. In contrast, when each dimension was analyzed separately using the /pa-ta-ka/ task, we found that prosody had the strongest contribution to discrimination accuracy. We believe that such behavior is associated with hyperkinetic dysarthria in ET patients due to the presence of uncontrolled movements in a controlled task. This deficit is likely to manifest as an excessive pitch, increased loudness variations, and decreased temporal regularity, which is best modeled by prosody with high-level features, such as the ones based on the fundamental frequency (*F*_0_) contour, energy, and duration, compared to articulatory features. However, future research is necessary to further extend and validate the methodological approaches on how to differentiate between differing diseases and dysarthria types.

When healthy speakers were included in the classification, we found that a fusion of articulation, phonation, and prosody supervectors also yielded the best performance for the monologue task, with an accuracy of up to 63% for the tri-class classification problem. However, in the case of the /pa-ta-ka/ task, only the prosody dimension achieved the best performance with accuracies of up to 71%. Once again, the prosody dimension had a fundamental role in discriminating between both pathologies and healthy speech, supporting the aforementioned results. When each dimension was analyzed separately using the monologue task, we observed that, on average, the phonation was the most discriminating because it requires accurate control of the vocal cords. We believe that this is the reason why it played such an important role in the tri-class classification problem. In addition, it is important to mention that most errors were due to the misclassification of PD patients as HC subjects. This is likely because of the greater overlap between characteristic speech changes associated with hypokinetic dysarthria and healthy aging compared to changes associated with hyperkinetic dysarthria. For instance, decreased voice quality, which is typical for PD patients^23^, is not uncommon in aging patients^24^. Conversely, pitch fluctuations are very specific to hyperkinetic dysarthria^7^, and to the best of our knowledge have never been documented in healthy aging speech.

Another relevant point to discuss is that two different tasks were evaluated in this study: the first one corresponds to a diadochokinetic exercise (i.e., /pa-ta-ka/ task) in which the articulatory muscles in charge of producing speech are required to be placed in specific positions at a very specific point in time; in other words, this is a controlled and functional task. The second one was a spontaneous speech task, in this case a monologue. This task can be captured unobtrusively and does not require the patient to perform specific movements using their articulators. The difference in the performance of these two tasks when attempting to distinguish between the two types of dysarthria is only 5%, which is still an excellent result, especially considering that evaluation through natural, connected speech may represent a very natural digital biomarker for the early diagnosis of diseases with similar clinical manifestations based on data acquired with minimal time cost or burden to the patient and investigator. Furthermore, considering that it is a language-dependent task, the robustness of the proposed methodology was shown with slightly lower results compared to the language-independent task (/pa-ta-ka/). In addition, the better performance of the diadochokinetic task in differentiating between PD and ET patients compared to the monologue task can likely be explained by the fact that ET patients often have issues affecting their cerebellum^25^. It is well known that cerebellar ataxia causes problems with the sequential planning needed for oral diadochokinetic tasks^26^.

It is also important to highlight the use of different datasets to create the UBMs. The two experiments described in this study exhibited their best performances when using UBMs derived from the Verbmobil (German) database. This may be because Czech (Slavic language) and German (Germanic language) are more closely related than Czech and Spanish (Romance language)^27^. Additionally, it is also interesting to note that the results did not improve when the Verbmobil (German) and CIEMPIESS (Spanish) corpora were combined to create a larger UBM; this suggests that the absolute volume of data is less important than collecting data of the appropriate language. In the case of the /pa-ta-ka/ task, we believe that linguistic similarity may not have as much of an impact since it is a language-independent task; consequently, this may explain why the best results in the tri-class classification problem were obtained from the UBM model trained on a combination of German and Spanish datasets, as this would result in a more generalizable model. However, further research is required before stronger conclusions can be made.

In addition to the differences in languages, the UBMs were trained using datasets with different recording procedures, resulting in differences in acoustic conditions and microphone types; however, the adaptation of the UBMs to the Czech GMMs was shown to be robust to these variables, allowing us to conclude that a methodology based on GMM-UBMs could be feasible even in corpora recorded under different conditions, something that has already been demonstrated mainly in speaker verification^28^. Nevertheless, it is important to stress that the Czech data were recorded using a professional head-mounted microphone in an environment with low ambient noise following international guidelines^7^, i.e., generally in better conditions than the training data. Therefore, we cannot exclude that the same robust results will be obtained when data for the evaluation would be in worse conditions (e.g., low-quality microphones, noisy environment, unbalanced distances, and different microphone positions) than training data. Therefore, future work could also consider evaluating different conditions in the target data, i.e., Czech speakers in our approach.

Finally, this work has some limitations. Although the UBM models trained with patients were not satisfactory, in this work, it was not possible to evaluate the scenario where a base model was trained with both pathologies because a corpus of ET speakers in other languages was not available, and taking part in it to create another UBM would considerably reduce the data to be evaluated, besides generating an imbalance between the Czech databases. However, with respect to UBM models trained with PD patients, we consider that these models can satisfactorily generalize ET because the PD patients used for training in Spanish and German languages^29,30^ were in moderate to advanced stages with potential occurrence of dyskinesias (i.e., introducing hyperkinetic speech behavior), which generates a feasible universe for an adaptation of the GMMs. Therefore, in future work, we consider it necessary to include more patient data to model in the UBM the large variability introduced by patients due to their patterns resulting from dysarthric symptoms. We also acknowledge that we did not perform specific testing for cognitive involvement or education, as the primary aim was the investigation of motor speech deviations. While the effect of cognitive impairment on motor speech, especially in ET patients, remains unknown, a recent study showed that cognitive impairment associated with PD may account for the worse performance of patients in tasks requiring temporal coordination, such as prolonged voicing, pause intervals, and decreased rate^31^. Since timing abnormalities in ET, such as low speech rates, are caused by the disease itself^9^, we believe that the potential occurrence of cognitive impairment in ET would have little impact on timing features.

Another limitation is that the duration of symptoms for ET patients is considerably longer than that of PD patients. This is primarily because some ET patients self-reported the occurrence of first motor symptoms already in childhood. Nevertheless, on average, perceptual speech impairment is comparable between PD and ET groups. Our approach was able to discriminate between ET and PD patients regardless of the duration of symptoms in the patients. Indeed, Spearman’s correlation test performed on the scores of ET patients and the duration of their symptoms did not reveal any significant correlations, suggesting that the duration of self-reported symptoms does not play a major role in the ability of speech assessment tools to distinguish between PD and ET patients. Indeed, speech production does not necessarily reflect disease duration or deteriorate at the same rate as other motor skills like gait or hand movement. Within our PD cohort, we did not find differing motor severities between those who had been correctly and incorrectly classified as PD. This is consistent with a previous multicentric study on PD showing that speech impairment severity was a non-overlapping marker of disease severity compared to other gross motor symptoms^21^. Finally, although our ET and PD groups were well-balanced with respect to age and gender, the majority of used acoustic features were treated by DC-level removal, amplitude normalization, and Z-score normalization. Future research may benefit from normalizing *F*_0_ from Hz to a semitone scale to avoid potential physical differences among speakers^32^.

In conclusion, we created GMM Supervectors with features extracted from three speech dimensions: articulation, phonation, and prosody, to distinguish between PD, ET, and healthy speech. The results showed that a fusion of the speech dimensions yielded the best results when applied to the bi-class classification problem, with an accuracy of 81.4 and 86.2% for the monologue and /pa-ta-ka/ tasks, respectively. In the tri-class classification problem (i.e., when healthy speakers were added as an additional class), the best result was obtained using a prosody-only model based on the /pa-ta-ka/ task (accuracy: 71.6%). The best result obtained from the monologue tasks was obtained from a combination of the three speech dimensions (accuracy: 63.3%). These results suggest that prosody and articulation are the two best-performing biomarkers for the differential diagnosis between PD and ET patients. Articulation features model the rigidity of the muscles involved in speech production, particularly during the transition between voiced and unvoiced sounds, while prosody models change in intonation, timing, and loudness. Future research is required to validate and extend our approach, especially for earlier stages of the disease, the use of deep learning architectures, and transfer learning strategies between languages and different types of dysarthria.

## Methods

**Fig. 4:**
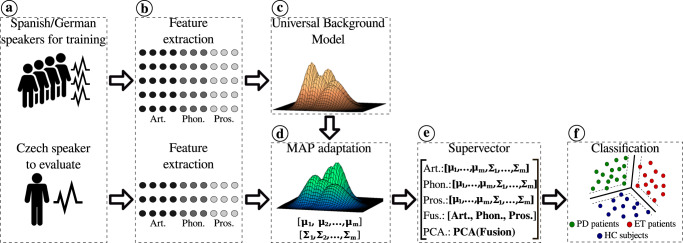
General methodology. **a** Databases considered. **b** Feature extraction. **c** UBM training. **d** Speaker adaptation. **e** Generation of supervectors. **f** Training and evaluation. GMM supervectors were created with information extracted from features of articulation (Art.), phonation (Phon.), and prosody (Pros.). Fus early fusion of all supervectors. PCA principal component analysis computed from the early fusion supervector. MAP maximum a posterior.

The methodology proposed in this work consists of six main stages: databases considered in this work (Fig. 4a). Extraction of articulation, phonation, and prosody features from each group of speakers (Fig. 4b). Training of the UBM (Fig. 4c). Adaptation of each speaker from the Czech corpora is performed using the Maximum a posteriori (MAP) method to derive a specific GMM per subject (Fig. 4d). Supervectors are created using the mean vectors and covariance matrices of the adapted GMM per subject (Fig. 4e). Training and evaluation of the Czech subjects is performed using a SVM classifier and following a cross-validation strategy. Bi-class and tri-class classification scenarios are considered (Fig. 4f). The details of each stage of the methodology are presented below.

### Data

The data considered in this study is divided into two main parts. The first part, called participants and speech recordings, provides information about the individuals who evaluated the proposed methodology. All participants in this evaluation spoke the same language (Czech). The second part pertains to the databases used to train the methodology, detailing the corpora utilized for training the UBM models. It should be noted that two different languages (Spanish and German) were used in the training process.

#### Participants and speech recordings

The participants were composed of several different groups of speakers, including 50 patients with ET (20 females), and 50 patients with PD (20 females), all of whom were Czech native speakers. The diagnosis of ET was established by previously published clinical research criteria^33^, while the PD diagnoses followed clinical diagnostic criteria outlined by the Movement Disorders Society^34^. Speech recordings were performed in a quiet room using a head-mounted condenser microphone (Beyerdynamic Opus 55) placed ~5 cm from the corner of the subject’s mouth. The recording procedure was performed according to dysarthria guidelines^7^. All speech signals were downsampled to 16 kHz. Symptom durations were estimated based on the self-reported occurrence of the first motor symptoms. All PD patients were in ON-state during the recording session, i.e., under the effect of dopaminergic medication. Each patient was evaluated by a neurological expert according to the third section of the Unified Parkinson’s Disease Rating Scale (UPDRS-III)^35^, which ranges between 0-108, our PD cohort had an average score of 20.1. The ET patients were evaluated by a neurological expert according to the Tremor research group Essential Tremor Rating Assessment Scale (TETRAS)^36^, which ranges between 0–64; the average score of the ET patients was 34.6. To identify any potential biases between the age of the patients and the severity of the disorder (measured using UPDRS-III and TETRAS for PD and ET, respectively), we obtained a Pearson’s correlation coefficient for each pathology. The results showed that there was no strong correlation between either group (PD patients: *r* = 0.233, *p* value = 0.10; ET patients: *r* = 0.036, *p* value = 0.81). Table 3 summarizes the subject’s demographic information.

**Table 3:** Demographic information of the speakers in the corpora considered to evaluate the proposed approach

Clinical characteristics	PD patients (*n* = 50; 30 men)	ET patients (*n* = 50; 30 men)	HC subjects (*n* = 50; 30 men)	*p* value
Age (years)	63.4 (9.5; 41–82)	64.8 (12.5; 31–82)	61.6 (11.2; 40–79)	0.21^a^;0.17^b^
Symptom duration (years)	6.7 (4.7; 0.7–24)	32.7 (17.2; 9–69)		n/a
UPDRS-III/TETRAS	20.1 (10.9; 4–54)	34.6 (15.8; 6–74)		n/a
UPDRS-III speech item	0.8 (0.6; 0–2)	0.7 (0.9; 0–3)	0.1 (0.3; 0–1)	<0.001^a^;0.08^b^

Values are listed in the format mean (standard deviation; range).

*PD* Parkinson’s disease, *ET* essential tremor, *HC* healthy control, *UPDRS-III* unified Parkinson’s disease rating scale—Third section, *TETRAS* tremor research group essential tremor rating assessment scale, *n/a* not applicable.

^a^Kruskal–Wallis test: PD patients vs. ET patients vs. HC subjects.

^b^ Mann–Whitney *U*-test: PD patients vs. ET patients.

In addition, data from 50 HC subjects (20 females) with ages 61.6 ± 11.2, ranging from 40 to 79 were included. None of the HC participants had a history of neurological or communication disorders. Each subject had at least eight years of elementary education. No participant exhibited severe intellectual impairments that would interfere with the study protocol.

Concerning the tasks considered in this study, we include two: the rapid repetition of the syllables /pa-ta-ka/ and a monologue. For the /pa-ta-ka/ task, the participants were instructed to perform rapid /pa/-/ta/-/ka/ syllable repetition at least seven times in a single breath. For the monologue task, participants were instructed to speak spontaneously for approximately 90 seconds about a freely chosen topic, which could be anything from hobbies, work, holidays, their hometowns, or a description of the current day. The participants were recommended to speak for ~90 s; no time limit was imposed. These two tasks were chosen for the following reasons: the /pa-ta-ka/ task is representative of functional vocal tasks that are essential for motor speech disorder assessment^7,9^; specifically, it tests the specific movements required to produce stop consonants with differing placement of articulators, while the monologue task represents the natural, unobstructed spontaneous speech production without any specific requirements. The average duration of /pa-ta-ka/ task was 7.8 ± 3.4 s for PD patients, 7.4 ± 2.4 s for ET patients, and 7.7 ± 4.2 s for the HC subjects. The average duration for the monologue task was 144 ± 56 s for PD patients, 117 ± 20 s for ET patients, and 150 ± 51 s for the HC subjects.

#### Databases considered to train the methodology

We used a variety of language databases to train the models according to the proposed methodology. Two corpora were related to the target phenomenon, in this case, the Parkinson’s database. Table 4 summarizes the clinical and demographic information of the participants. We also included two other spontaneous speech corpora which are typically used for training speech recognition systems. We included them to improve the training of our system and also considering that several works have performed experiments with these databases and showed that they could be useful to create models of pathological speech^37,38^. More details on each database are given below.

**Table 4:** Demographic information of the speakers in the corpora considered in the UBM training

Clinical characteristics	PD patients	HC subjects	*p* value
**Spanish**	*n* = 50; 25 men	*n* = 50; 25 men	
Age (years)	61.0 (9.4; 33–81)	60.9 (9.4; 31–86)	0.49^a^
Symptom duration (years)	10.6 (9.2; 1–43)		n/a
MDS-UPDRS-III	36.5 (16.5; 6–75)		n/a
MDS-UPDRS-III speech item	1.3 (0.8; 0–3)	0.2 (0.4; 0–1)	<0.001^a^
**German**	*n* = 88; 47 men	*n* = 88; 44 men	
Age (years)	66.5 (8.9; 42–84)	63.2 (13.9; 26–85)	0.15^a^
Symptom duration (years)	7.1 (5.9; 1–30)		n/a
UPDRS-III	22.7 (10.9; 5–55)		n/a
UPDRS-III speech item	1.3 (0.6; 0–3)	0.1 (0.3; 0–1)	<0.001^a^

Values are listed in the format mean (standard deviation; range).

*PD* Parkinson’s disease, *HC* healthy control, *MDS-UPDRS-III* movement disorders society—unified Parkinson’s disease rating scale—Third section, *UPDRS-III* unified Parkinson’s disease rating scale—Third section, *n/a* not applicable.

^a^Mann–Whitney *U*-test: PD patients vs. HC subjects.

**PD-Spanish:** PC-GITA contains the recordings of 50 PD patients and 50 HC subjects, all of whom were native speakers of Colombian Spanish^29^. The patients in the PC-GITA database were evaluated by an expert neurologist and labeled according to the third section of the Movement Disorders Society—Unified Parkinson’s Disease Rating Scale (MDS-UPDRS-III)^39^, with an average of 36.6 points. All patients were in ON-state during the recording session.

**PD-German:** This corpus consists of 88 PD patients and 88 HC subjects, all of whom were German native speakers^30^. Similar to PC-GITA, the patients were in ON-state during the recording session and were evaluated according to the UPDRS-III scale. German patients had an average of 22.7 on the UPDRS-III score. The same tasks described in the previous section were also considered in the German and PC-GITA databases.

**CIEMPIESS:** This corpus consists of 17 h of FM podcasts in Mexican Spanish^40^. The data comprises 16,717 audio files produced by a total of 96 male and 45 female speakers. The samples were recorded at a sampling frequency of 16 kHz with 16-bit resolution.

**Verbmobil:** This corpus consists of speech recordings of 586 German native speakers (278 female) for a total of 29 h of speech. The data comprises 11,714 audio files recorded at a sampling frequency of 16 kHz with 16-bit resolution. Each recording was collected in a controlled acoustic environment using a close-talk microphone^41^.

#### Ethical approval and informed consent

All participants provided written informed consent prior to their inclusion. For the collection of the corpus from Czech speakers, the study received approval from the ethics committee of the General University Hospital in Prague, Czech Republic. The databases used to train the methodology were approved by the ethical research committee of the University of Antioquia, Colombia (PD-Spanish) and by the ethics committee of the Ruhr University of Bochum, Germany (PD-German). All procedures were performed following the ethical principles laid down by the Declaration of Helsinki.

### Feature extraction

Articulation, phonation, and prosody features were extracted to model different speech deficits in subjects suffering from motor speech disorders such as those associated with PD or ET. For this stage, a DC offset removal and amplitude normalization were performed on each recording; this improved the robustness of the processing and ensured that the signals were at a suitable scale for characterization. 78 features (7 phonatory, 58 articulatory, and 13 prosodic) were extracted using the DisVoice toolkit^42^, the definition of each feature is summarized in Table 5. Details of each speech dimension are presented below.

**Table 5:** Overview of applied speech features

Feature	Unit	Dimension	Description
Bark band energies	-	Articulation	Twenty-two Bark band energies in onset transitions
MFCCs	-	Articulation	Twelve Mel frequency cepstral coefficients in onset transitions
Δ - ΔΔ MFCCs	-	Articulation	First and second derivative of the MFCCs in onset transitions
Δ- ΔΔ*F*_0_	Hz	Phonation	First and second derivative of the fundamental frequency
Jitter	%	Phonation	Average absolute difference between consecutive periods, divided by the average period.
Shimmer	%	Phonation	Average absolute difference between the amplitudes of consecutive periods, divided by the average amplitude.
APQ	%	Phonation	Eleven-point amplitude perturbation quotient, the average absolute difference between the amplitude of a period and the average of the amplitudes of it and its ten closest neighbors, divided by the average amplitude.
PPQ	%	Phonation	Five-point period perturbation quotient, the average absolute difference between a period and the average of it and its four closest neighbors, divided by the average period.
Energy	dB	Phonation	Energy of voiced segment
Voiced segment	s	Prosody	Duration of the voiced segment
Model *F*_0_ contour	-	Prosody	Coefficients of 5-degree Lagrange polynomial to model *F*_0_ contour
Model energy contour	-	Prosody	Coefficients of 5-degree Lagrange polynomial to model energy contour

#### Articulation

This speech dimension evaluates the ability of a speaker to control the movement of their articulators such that they are in the correct position at the correct time, and held for the appropriate duration and energy while producing speech. This study used the transition from unvoiced to voiced segments (onset) to assess the difficulties that speakers suffering from dysarthria had with starting the vibration of the vocal folds^22,43^. We did not include the transition from voiced to unvoiced segments (offset) because previous work has shown that onset transitions exhibit better or equal performances compared to offset transitions or a combination of both^44^. Onset transitions were segmented according to the presence of the *F*_0_, which was estimated using Praat^45^. Once the borders are detected, 40 ms of the signal are taken to the left and to the right of each border, forming segments with 80 ms length^22,43,46^. A total of 58 features were extracted from the transition segments, including the energy content in 22 critical bands distributed according to the Bark scale, and 12 Mel frequency cepstral coefficients together with their first and second derivatives. The features were computed at the frame-level in each onset segment upon windows with 40 ms length and a time-shift of 20 ms. Additional information and the source code can be found in^42^^,^^47^.

#### Phonation

This speech dimension models the ability of a speaker to use air in their lungs to make their vocal folds vibrate, allowing for the production of voiced sounds. In this paper, we focused mainly on the production of voiced sounds to model the ability of subjects to control their vocal fold vibration. The phonation feature set was composed of seven measures computed exclusively over voiced segments of the speech signal: (1,2) the first and second *F*_0_ derivatives; (3) shimmer, which measures amplitude perturbation within three consecutive cycles of the vocal folds’ vibration; (4) jitter, which measures frequency perturbation in three cycles of the vocal folds’ vibration; (5,6) the amplitude and pitch perturbation quotients, namely APQ and PPQ, respectively, which model the long-term amplitude and temporal variation in the vibration cycles of the vocal folds; and (7) the log energy per frame as an indirect measure of loudness. Additional information about the computation of phonation features is presented in^47^^,^^48^.

#### Prosody

This dimension measures the ability of a speaker to produce and control changes in intonation, timing, and loudness. A total of 13 prosody features were extracted from each voiced segment, including the duration of the segment, the coefficients of a fifth-order polynomial that models the *F*_0_ contour, and the coefficients of a 5th-order Lagrange polynomial that models the energy contour. Additional information about this approach to model prosodic information can be found in^49^.

### Gaussian mixture models—universal background models

The dynamics of the extracted features given by the variability in the extracted segments (transitions and voiced segments) for each audio sample were modeled using a GMM-UBM framework. GMMs are probability models that represent a population of a linear combination of Gaussian probability distributions. For a *D*-dimensional feature vector ***x***, where *D* = 58 for the articulation feature set, *D* = 7 for the phonation feature set, and *D* = 13 for the prosody feature set, the mixture density used for the likelihood function in *M* Gaussians is defined as $p({{{\boldsymbol{x}}}}| \lambda )=\mathop{\sum }\nolimits_{i = 1}^{M}{w}_{i}{p}_{i}({{{\boldsymbol{x}}}})$, where *p*_*i*_(***x***) corresponds to a Gaussian density weighted by *w*_*i*_ such that it satisfies the constraint $\mathop{\sum }\nolimits_{i = 1}^{M}{w}_{i}=1$. In addition, each *p*_*i*_ distribution is composed of a mean vector ${\left[{{{{\boldsymbol{\mu }}}}}_{i}\right]}_{D\times 1}$ and a covariance matrix ${\left[{{{{\boldsymbol{\Sigma }}}}}_{i}\right]}_{D\times D}$. The set of parameters for the density model is denoted as $\lambda =\left\{{w}_{i},{{{{\boldsymbol{\mu }}}}}_{i},{{{{\boldsymbol{\Sigma }}}}}_{i}\right\}$, where *i* = 1, …, *M*. The parameter set *λ* of the maximum likelihood function can be estimated using the expectation maximization (EM) algorithm^50^, which iteratively re-defines the parameters and increases the likelihood of the estimated model for the observed feature vectors; that is, for iterations *k* and *k* + 1, *p*(***X***∣*λ*^(*k*+1)^) > *p*(***X***∣*λ*^(*k*)^), where ***X*** is a matrix with the group of features ***x*** extracted from each participant for the different speech dimensions (articulation, phonation, and prosody)^51^.

### Maximum a posteriori adaptation

The parameters that model each target speaker were derived from an adaptation process denoted as maximum a posteriori (MAP)^52^. Unlike the use of the GMM and the EM algorithm, the MAP adaptation aims to derive parameter updates from UBMs trained using the Spanish and German databases. This approach is considered to be relatively robust, resulting in the generation of well-trained models that provide a closer coupling between each model and the UBM model. The process for the MAP adaptation is divided into two main steps: (1) the probability that a feature vector belongs to each Gaussian of the UBM is estimated; (2) new values for each parameter were estimated by taking into account the probability obtained in the previous step as well as the estimated parameters obtained from previous iterations of the adaptation process^51^.

Given a UBM and a matrix ${{{\boldsymbol{X}}}}=\left\{{{{{\boldsymbol{x}}}}}_{1},\ldots ,{{{{\boldsymbol{x}}}}}_{T}\right\}$ that contains *T* feature vectors, we first determine the probability of a feature vector to belong to the *i*th Gaussian as shown in Equation (1).

1 $$Pr(i| {{{{\boldsymbol{x}}}}}_{t})=\frac{{w}_{i}{p}_{i}({{{{\boldsymbol{x}}}}}_{t})}{\mathop{\sum }\nolimits_{j = 1}^{M}{w}_{j}{p}_{j}({{{{\boldsymbol{x}}}}}_{t})}$$

Then, we use *Pr*(*i*∣***x***_*t*_) and ***x***_*t*_ to calculate the statistics denoted by *n*_*i*_, *E*_*i*_(***x***), and *E*_*i*_(***x***^2^) that allow finding the parameters *λ*.

2 $${n}_{i}=\mathop{\sum }\limits_{t=1}^{T}Pr(i| {{{{\boldsymbol{x}}}}}_{t})$$

3 $${E}_{i}({{{\boldsymbol{x}}}})=\frac{1}{{n}_{i}}\mathop{\sum }\limits_{t=1}^{T}Pr(i| {{{{\boldsymbol{x}}}}}_{t}){{{{\boldsymbol{x}}}}}_{t}$$

4 $${E}_{i}({{{{\boldsymbol{x}}}}}^{2})=\frac{1}{{n}_{i}}\mathop{\sum }\limits_{t=1}^{T}Pr(i| {{{{\boldsymbol{x}}}}}_{t}){{{\rm{diag}}}}({{{{\boldsymbol{x}}}}}_{t}{{{{\boldsymbol{x}}}}}_{t}^{{\mathsf{T}}})$$

Finally, from the calculated statistics the parameters ${w}_{i}^{{\prime} }$, ${{{{\boldsymbol{\mu }}}}}_{i}^{{\prime} }$, and ${{{{\boldsymbol{\Sigma }}}}}_{i}^{{\prime} }$ are updated for the *i*th Gaussian mixture using the following equations:

5 $${w}_{i}^{{\prime} }=\left[{\alpha }_{i}{n}_{i}/T+(1-{\alpha }_{i}){w}_{i}\right]\gamma$$

6 $${{{{\boldsymbol{\mu }}}}}_{i}^{{\prime} }={\alpha }_{i}{E}_{i}({{{\boldsymbol{x}}}})+(1-{\alpha }_{i}){{{{\boldsymbol{\mu }}}}}_{i}$$

7 $${{{{\boldsymbol{\Sigma }}}}}_{i}^{{\prime} 2}={\alpha }_{i}{E}_{i}({{{{\boldsymbol{x}}}}}^{2})+(1-{\alpha }_{i})({{{{\boldsymbol{\Sigma }}}}}_{i}^{2}+{{{{\boldsymbol{\mu }}}}}_{i}^{2})-{{{{\boldsymbol{\mu }}}}}_{i}^{{\prime} 2}$$

Where *γ* is a scale factor that guarantees $\mathop{\sum }\nolimits_{i = 1}^{M}{w}_{i}^{{\prime} }=1$. Also, *α*_*i*_ is known as the adaptive coefficient that controls the balance between old and new parameters, and it is computed as ${\alpha }_{i}=\frac{{n}_{i}}{{n}_{i}\,+\,r}$, where *r* is a relevance factor that has been defined in the literature as a standard term equivalent to 16^51^.

### Supervectors

A GMM supervector can be considered as a representation in smaller-dimensional vectors after adaptation from the UBM. This new representation summarizes the dynamic information contained in each temporal feature (segments) in a more compact format, generating a comprehensive static representation for each recording; this has the advantage of providing statistical information of the phenomenon; i.e., a mean vector and a covariance matrix. For this work, GMM supervectors were created by stacking the means ${{{{\boldsymbol{\mu }}}}}_{i}^{{\prime} }$ and the diagonal of the covariance matrix ${{{{\boldsymbol{\Sigma }}}}}_{i}^{{\prime} }$ derived from the mixture components. In this case, both statistics have the same dimension and are determined by the product of the number of Gaussian components *M* and the number of features in each speech dimension.

### Parameters optimization and classification

We used an SVM classifier with a Gaussian kernel with each hyperparameter optimized using a grid-search such that *C* ∈ {0.001, 0.005, 0.01,⋯, 100, 500, 1000} and *γ*_*k*_ ∈ {0.0001, 0.001, ⋯, 1000}. Note that the optimal hyper-parameters were obtained during the training process as the mode over the repetitions. Each experiment was trained and evaluated following a stratified k-fold cross-validation strategy with ten folds. This process was repeated ten times for a better generalization of the results. We also performed an adaptation for different numbers of Gaussian components *M* ∈ {2, 4, 8, 16, 32, 64, 128}. Results only show the number of Gaussians that yielded the highest accuracy. Accuracy was used to evaluate the methodology. Sensitivity and specificity are reported in the experiments with the best results to allow further analyses regards false positives and false negatives.

### Reporting summary

Further information on research design is available in the Nature Research Reporting Summary linked to this article.

### Supplementary information

Reporting Summary

## Data Availability

Individual participant data that underlie the findings of this study are available upon reasonable request from the corresponding author. The data are not publicly available due to their containing of information that could compromise the privacy of study participants.

## Code availability

Feature extraction was performed using the DisVoice toolkit, a Python framework designed to compute features from speech files and can be accessed via this link.
